# Sparse Annotation is Sufficient for Bootstrapping Dense Segmentation

**DOI:** 10.21203/rs.3.rs-5339143/v1

**Published:** 2024-11-14

**Authors:** Vijay Venu Thiyagarajan, Arlo Sheridan, Kristen M. Harris, Uri Manor

**Affiliations:** 1 Department of Neuroscience, Center for Learning and Memory, University of Texas at Austin, Austin Texas, 78712; 2 Waitt Advanced Biophotonics Center, Salk Institute for Biological Studies, La Jolla, CA, 92037; 3 Department of Cell & Developmental Biology, School of Biological Sciences, University of California, San Diego, CA 92093; 4 Halıcıoğlu Data Science Institute, University of California, San Diego, CA 92093

## Abstract

Producing dense 3D reconstructions from biological imaging data is a challenging instance segmentation task that requires significant ground-truth training data for effective and accurate deep learning-based models. Generating training data requires intense human effort to annotate each instance of an object across serial section images. Our focus is on the especially complicated brain neuropil, comprising an extensive interdigitation of dendritic, axonal, and glial processes visualized through serial section electron microscopy. We developed a novel deep learning-based method to generate dense 3D segmentations rapidly from sparse 2D annotations of a few objects on single sections. Models trained on the rapidly generated segmentations achieved similar accuracy as those trained on expert dense ground-truth annotations. Human time to generate annotations was reduced by three orders of magnitude and could be produced by non-expert annotators. This capability will democratize generation of training data for large image volumes needed to achieve brain circuits and measures of circuit strengths.

In 3D instance segmentation, voxels of a 3D image volume are partitioned into distinct object instances, where each voxel is associated with its corresponding object of interest. Instance segmentations of brain neuropil comprising intricate processes of dendrites, axons, and glia, are required for downstream connectomics and ultrastructural studies^1,2^. The voxel assignment in neuropil segmentation is often challenging due to the complex morphologies of the fine processes, which often branch and converge at multiple locations in the volume. Individual flaws in a segmentation can have large consequences in the resultant topology of the segmented processes. For example, if an axonal process were to be accidentally connected at a branch point to a different axon, the resulting connection could project to the wrong target neurons.

Despite the challenges, automatic deep learning-based methods for electron microscopy (EM) segmentation have shown great promise^1–7^. Flood-Filling Networks (FFN) are currently considered to be state-of-the-art^6^; however, the computational resources required to use and train FFNs are unattainable for most laboratories. A different approach involves convolutional neural networks to generate boundary predictions on image volumes, followed by prediction and post-processing to output a 3D instance segmentation^3–5,7,8^. This boundary-based approach requires 100x less computational cost and is easily parallelized, enabling much faster processing of large volumes, but it is usually less accurate than FFNs^5^. Recent work showed that boundary detection-based methods can match the accuracy of FFNs with 10–100x higher efficiency by adding local shape descriptors (LSDs) for auxiliary training^7^. Additionally, there are other efficient methods to generate large segmentations without LSDs, such as boundary predictions with improved post-processing using mutex watershed or multicut^9,10^. Thus, the implementation of efficient methods provides an important step towards acceleration of 3D segmentation.

The performance of deep learning models is fundamentally dependent on the quality and quantity of the available training data. For segmentation of brain neuropil, ground-truth data typically must be dense and diverse to be useful. Explicitly, all objects in a volume must be completely annotated (dense) and sampled from multiple (diverse), representative regions of the target data. Previous studies regarding the accuracy of different deep learning methods used large amounts of ground-truth data for training and evaluation. The annotations in these training data required enormous human effort. For example, the zebra finch EM dataset had 33 densely annotated training volumes, for a total of ~200μm^3^ of labeled objects, and 50 manually proofread skeletons totaling 97 mm^6,7^. Obtaining this ground-truth dataset required multiple time-consuming refinement rounds by expert annotators (Joergen Kornfeld, personal communication). In another example, the complete morphological reconstruction of 15 Kenyon cells in the adult fly brain, each with a mean cable length of 0.78 mm, took more than 150 human hours^11^. The annotated ground-truth for every dendritic, axonal, and glial process in a rat hippocampal volume of 180μm^3^ took more than 1,000 hours of manual annotation and curation^12^. These examples illustrate that the human effort spent generating ground-truth data currently stands as a major bottleneck in the production of useful segmentations and downstream analyses.

We present a new 2D→3D approach that markedly reduces the human effort to generate ground-truth data that can be used to improve machine learning algorithms for automated 3D segmentation. We evaluated the 3D segmentations that were generated as a function of the amount of annotations and found similar accuracy for all amounts, including as little as ten minutes of non-expert annotations on a single image. We also tested whether the generated 2D→3D segmentations can be used without any proofreading or curation as pseudo ground-truth training data for bootstrapping 3D models. We show the method worked for multiple brain neuropil datasets as well as a fluorescence microscopy dataset, thus illustrating its generalizability. We provide a workflow for new users that helps to replace the enormous human effort currently needed to trace and curate experimental 3D electron microscopy datasets. We anticipate that the new tools and techniques will enable most laboratories to generate training data for complex 3D instance segmentation tasks rapidly, even if they lack the otherwise prohibitive compute resources or human expertise.

## Results

**Fig. 1 |:**
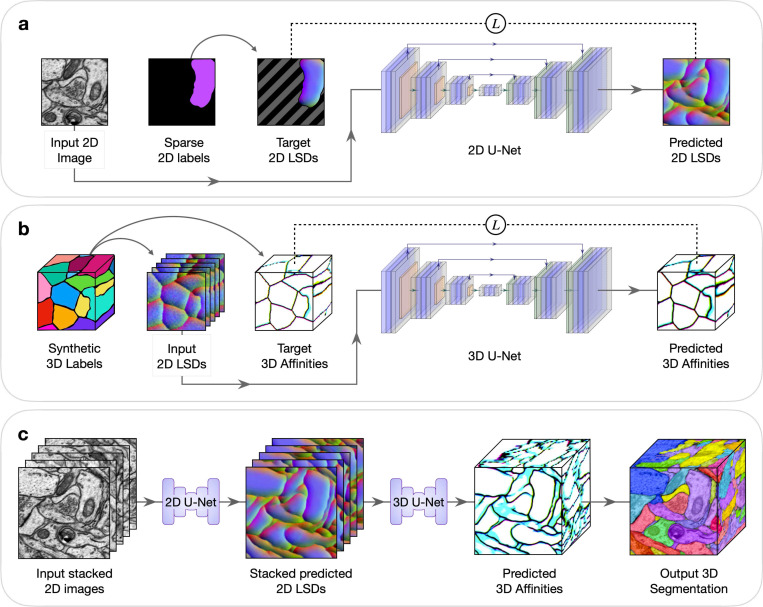
2D→3D Method to generate dense 3D instance segmentations from sparse 2D training data. Five example sections are shown in stacked 2D images or LSDs to simplify visualization. LSDs show the first three components as RGB-channels. All networks compute a weighted mean-squared-error (MSE) loss between the predictions and targets during training, denoted by L. **a**, Sparse 2D to dense 2D. Input 2D images with sparse labels are used to train a 2D U-Net to learn dense 2D LSDs. Background regions of the target 2D LSDs, denoted by diagonal gray stripes, are not used in the computation of the loss during training. **b**, Stacked dense 2D to dense 3D. A 3D U-Net is trained to learn 3D affinities from stacked 2D LSDs using synthetic 3D labels. **c**, Combined 2D to 3D inference pipeline. Sections from a 3D image volume are used as input to the trained 2D U-Net to generate stacked 2D LSDs. The trained 3D U-Net infers 3D affinities from the stacked 2D LSDs. A 3D segmentation is generated from the 3D affinities using standard post-processing techniques.

Deep learning networks can learn to generate dense 3D volumetric predictions from sparsely annotated 2D slices^13^. However, this approach has not yet been extended to highly anisotropic data or to complex 3D instance segmentation that are typical of serial section EM datasets. To tackle this, we have developed an approach in which a human first produces sparse 2D annotations on an image (or subset of images). Next, a 2D network is trained on the sparse 2D annotations to make dense predictions on each image (Fig. 1a). Then these 2D predictions are stacked spatially as input for a separate lightweight 3D network, trained using random synthetic 3D data generated on the fly, to infer 3D boundaries from the 2D stacks (Fig. 1b). Finally, conventional post-processing is done to obtain a 3D segmentation (Fig. 1c).

Using this new approach, we present experimental results of bootstrapping neuropil segmentation models with pseudo ground-truth training data generated rapidly by the 2D→3D method. The segmentation models were trained on various amounts and types of sparse annotation. We compared the quality and accuracy of segmentations generated by 3D LSD models bootstrapped from different amounts and types of annotation. We also compared the total time to generate the segmentations for each level of sparse manual annotation.

### Datasets

We chose six datasets for these experiments, including HARRIS-15^12^, FIB-25^14^, CREMI-A,B,C^15^, and EPI^16^. Each dataset (except EPI) consisted of two EM volumes with dense annotations, Volume 1 and Volume 2, with dataset names, content descriptions, sizes, number of instances, imaging modalities, and resolutions detailed in Supplementary Table 1. At least two volumes per dataset were required to avoid propagating volume-specific biases. To show this approach extends beyond EM, we included confocal laser scanning microscopy volumes of plant ovules (EPI).

For every dataset, we performed instance-level ablations on the dense labels of Volume 1 to generate different amounts of sparse training data (Supplementary Fig. 1). We chose the following instance-level ablations: 1 object, 2 adjacent objects, 2, 5, 10, 50, or 100 random objects. For every ablation, we chose three mutually exclusive selections of the required number of instances. In addition, we spent 30 minutes to generate non-expert sparse 2D annotations in a single section and another 30 minutes for sparse 3D annotations across a few sections on Volume 1 for each dataset (Supplementary Fig. 2). The non-expert annotations were partitioned into three artificial 10-minute annotations such that labels were mutually exclusive. The different instance-level ablations and manual annotations, which we call ‘sparsities’, tested in the experiments are detailed in Supplementary Note A.

**Fig. 2 |:**
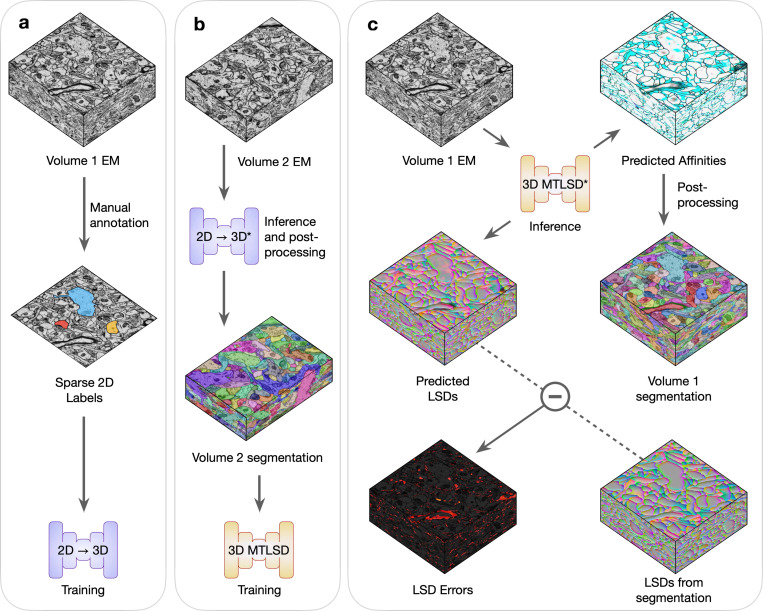
Bootstrapping dense 3D segmentations. Volumes 1 and 2 are two unannotated EM volumes. **a,** Sparse 2D ground-truth labels are manually created on Volume 1, which are used to train the 2D→3D networks. **b,** A 3D segmentation of Volume 2 is generated using the trained 2D→3D networks (denoted by *) and standard post-processing. The resulting 3D segmentation is used as pseudo ground-truth training data, without any masking or proofreading, for an untrained 3D MTLSD network. **c,** The trained 3D MTLSD network infers 3D affinities and LSDs on Volume 1. Post-processing is applied to the 3D affinities to generate a 3D segmentation on Volume 1, from which LSDs are computed. The element-wise difference between the model’s LSDs and the computed segmentation LSDs is used to visualize a heat-map of LSD prediction errors.

For every combination of dataset and initial training data, we conducted the experimental procedure as described in Fig. 2 and Supplementary Note B. Briefly, Volume 1’s sparse annotations were used to train the 2D→3D method and then segment Volume 2. The generated segmentation of Volume 2 was used to train a 3D model, which was then used to segment Volume 1. Inference and post-processing steps were conducted in a grid-fashion to explore a range of values for parameters that might influence the output segmentations. We designated the best performing segmentations in the grid as the representative bootstrapping results for that dataset and initial amount of training data.

To evaluate the accuracy of bootstrapped (Volume 1) segmentations as a function of the amount of initial training data, we report segmentation accuracy with the min-cut metric (MCM). The MCM is a skeleton-based measure of the total number of split and merge edit operations that would need to be proofread to ensure accuracy^7^. We adapt it to be the total number of edits needed divided by the total number of objects in the ground-truth. Ground-truth labels were filtered to remove labels smaller than 500 pixels and connected components were relabeled. Ground-truth skeletons were generated as described in Supplementary Note C. We report additional metrics such as variation of information in Supplementary Figures 4–13.

### Bootstrapped Segmentations

**Fig. 3 |:**
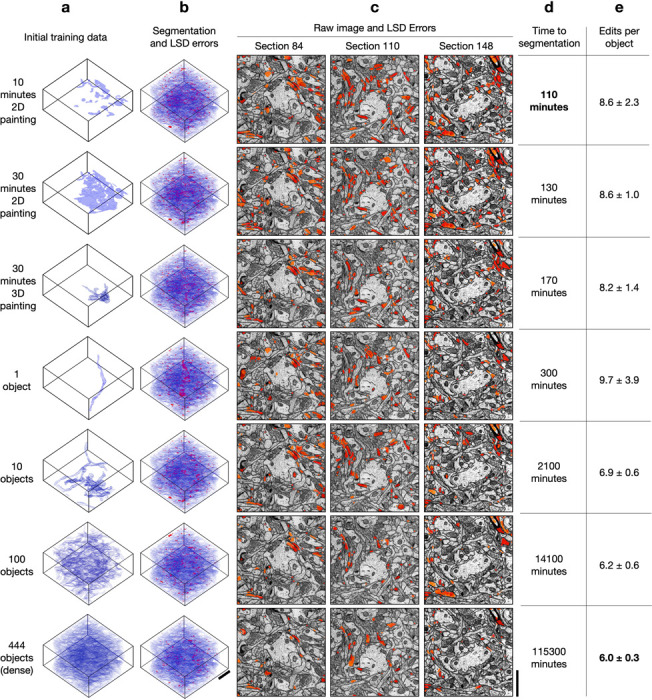
Bootstrapped models for HARRIS-15 3D segmentation. **a,** Initial ground-truth training data used for creating pseudo ground-truth (pGT). **b,** Bootstrapped segmentations from pGT showing agreement with GT (blue) and LSD split/merge errors (red). LSD Errors shown are the element-wise difference between the LSDs computed from dense GT and the LSDs computed from the bootstrapped segmentations. **c,** 2D sections of LSD errors overlaid on EM images. **d,** Total time for 3D segmentation, including manual annotation, training, and post-processing. **e,** Average number (± s.d.) of split and merge edits required per object to match ground-truth skeletons. Edits per object are shown for varying amounts of initial training data. Scale bars, 1.5 μm.

**Fig. 4 |:**
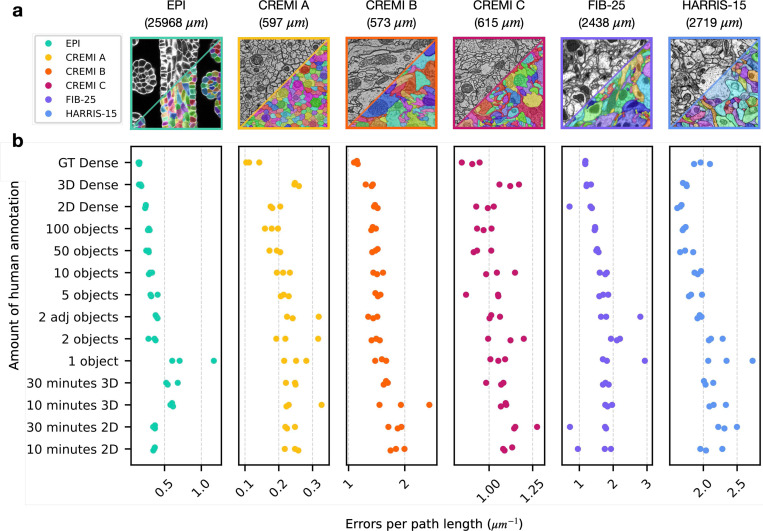
Quantitative results of 3D segmentations bootstrapped from sparse annotations. **a,** Example images and ground-truth labels from each dataset and color-coding key. The total path length of the dense ground-truth (GT) skeletons in each training volume is indicated in parenthesis. **b,** Number of split and merge errors needing correction per skeleton path length to match the dense GT skeletons. Lower scores are better. Each dataset had three separate tests for each amount of annotation (illustrated as three dots of the same color per row). Dense segmentations of a separate test volume (pseudo-GT) were first created by 2D→3D models trained on different amounts of initial GT annotations, which are sorted from bottom to top of the y-axis in order of increasing human annotation effort. Scores were then computed by comparing GT annotations to segmentations produced by a 3D model trained on the pseudo-GT segmentation. 2D (or 3D) Dense refers to training the initial 2D→3D (or 3D) model on all available 2D (or 3D) annotations to generate pseudo-GT. GT Dense refers to directly training a 3D model on manual GT annotations of the test volume, as opposed to pseudo-GT, to generate the bootstrapped segmentation.

For all datasets, we found the quality of pseudo ground-truth segmentations and bootstrapped segmentations to be good for all sparsities, including ten minutes of sparse 2D annotation (Fig. 3, Fig. 4, Supp. Fig. 4–13). Importantly, sparse non-expert annotations of a single section yielded bootstrapped segmentations similar in accuracy to dense expert annotations of the volume (Fig. 4). One possible explanation is that ten minutes of annotations in a single image contained enough boundary information between instances that the 2D network learned accurate 2D predictions with significantly less human annotation time.

We note that the quality of bootstrapped segmentations is dependent on the image data and the quality of the generated pseudo ground-truth used for training. The occurrence of high LSD error values along the boundaries of the segmented processes indicate minor pixel-wise differences with the boundaries of the manual ground-truth labels and not a significant difference in the morphology of the process. In Fig. 3, we apply binary opening to the LSD error mask and use it to visualize the LSD errors without the minor boundary differences. This operation is especially useful in the cases where manual annotations are not pixel-precise along boundaries.

We measured the total time to generate a segmentation for all sparsities by summing the estimated human annotation time with the time taken by the machine for all training, inference, and post-processing (Fig. 3). The machine time was ~100 minutes for all datasets and sparsities. A model bootstrapped with 10 minutes of sparse annotations on a single section was able to generate a dense 3D segmentation in about 110 minutes. In contrast, a model bootstrapped from dense annotations made with 1000x more human annotation time generated a segmentation requiring similar proofreading effort, with at most 2–3 fewer edits per path length in microns (Fig. 4). We conclude that densely annotated ground-truth is not necessary to provide a starting point for training dedicated 3D models. Instead, sparse annotations are sufficient for bootstrapping automatic neuropil segmentation and require about the same amount of proofreading for marked reduction in total time.

The new algorithms are available at GitHub (github.com/ucsdmanorlab/bootstrapper) to generate dense 3D segmentations from sparse 2D annotations and bootstrap 3D models. We implemented a Napari plugin to allow users to apply the 2D→3D method to generate and export dense volumetric segmentations from minimal annotations made through Napari’s graphical user interface (github.com/ucsdmanorlab/napari-bootstrapper). Our plugin, along with pre-trained models and plugins like Segment Anything Model (SAM) fine-tuned on microscopy data, can reduce manual 2D annotation to a few clicks^17,18^.

## Discussion

Machine learning models depend on sufficient quantities of high quality training data. Generating training data for segmentation of multidimensional datasets, e.g., 3D neuropil segmentation, is prohibitively time consuming to generate and proofread manually. This is largely due to the difficulty in segmenting 3D structures on digital display screens that can only display one 2D image at a time. These physical limitations coupled with the need for densely annotated data from a diverse range of example images that represent the full landscape of highly variable features renders 3D annotation a monumental task that most researchers and laboratories are not capable of within a reasonable timeframe. The total resulting cost of generating manually annotated data in both human hours and research dollars ultimately presents a barrier towards incalculable new discoveries. Thus, there is an urgent need to develop new tools and methods that can massively accelerate and democratize the generation of ground-truth data and automated segmentation.

Here we have demonstrated the novel use of a 2D→3D method to generate dense volume segmentations rapidly from sparse 2D annotations for complex instance segmentation tasks. We also demonstrated that rapidly generated segmentations provide sufficiently accurate pseudo ground-truth training data for bootstrapping 3D segmentation models. These lightweight models do not perform as well as a dedicated 3D model trained on dense and diverse ground-truth annotations. However, the 100–1000x shorter amount of time required to segment many complex subvolumes far outweighs the cost of training human annotators to produce accurate dense manual annotations for use in dedicated 3D models. Furthermore, the relative ease of refining a dedicated model trained on such subvolumes will ultimately result in automation of better large scale instance segmentations. In short, this approach enables massively accelerated convergence on ground-truth annotations at a fraction of the cost, and is effective for even the most complex instance segmentation tasks such as neuropil segmentation.

We hypothesize the 2D→3D method performs well because the provided sparse annotations capture enough important boundary information. However, the method is not robust to poor boundary annotations and highly anisotropic data where z-connectivity between boundaries is unclear even to humans. Despite these limitations, this approach eliminates the need to annotate objects section by section as a few sparse but critical boundary annotations are sufficient for bootstrapping.

Generating dense volumetric instance segmentations from sparse image annotations has been demonstrated for simpler biomedical image segmentation tasks by generalist algorithms^13,19–22^, but not for complex instance segmentation tasks like neuropil segmentation. Our method works reliably on small volumes for all initial amounts of sparse image annotations and all investigated datasets, suggesting general applicability. The use of pseudo, incomplete, or imprecise labels as training data is also not novel; neither is the idea of iterative bootstrapping to refine a model by acquiring more segmented data for training^23–26^. Others recently demonstrated an end-to-end pipeline that learned to correct swift and incomplete annotations like skeletons and seeds^27^, and labels^28^. Previous work has also addressed the dense annotation bottleneck with the use of pixel embeddings to produce sparse instance masks generated on the fly during training to achieve weak positive-unlabeled supervision^29^. Our use of LSDs enhances these approaches by quickly providing high error regions that streamline proofreading and facilitate automatic refinement of annotations. Thus, LSD-based models make quickly generating ground-truth a practical possibility for large 3D datasets.

In addition to the increased computational efficiency of LSD-based networks, the mask of high error regions from the LSD errors has many valuable applications. A natural application is its use in a targeted proofreading tool. Future work could guide the tool or user to locate potential errors^30–34^. Automatic error correction methods could also learn to correct errors from LSD errors and prompt the user for corrections that require a single click^27,35,36^. Additionally, the mask of high errors can be used for targeted refining of a model during bootstrapping; masked regions in the field of view can be ignored in the loss computation while forcing the model to encounter more FOVs from high error regions.

There are natural future directions for this work. An auto-context approach could be adopted for the 2D→3D method, where the raw EM volume along with the stacked 2D predictions would be inputs to the 3D network^37^. We hypothesize this modification would resolve some ambiguities when extrapolating connectivity between predictions of adjacent sections of the image volume. However, this modification would require dataset-specific training for the 3D network, more diverse and neuron-inspired synthetic labels for training the 3D network, and the use of long-range affinities and mutex watershed for segmentation^4,9^.

Together, the presented LSD-based bootstrapping method greatly democratizes the generation and use of annotation data for automated segmentation. This is but a step in the right direction—future work should continue to prioritize approaches that require minimal manual annotation. Self-supervised pre-training approaches that leverage the large amounts of available image data can be integrated into bootstrapping workflows to accelerate even further progress towards automated image segmentation and analysis, thereby enabling scientists to focus more on new discoveries and insights.

## Methods

The general principle is that 2D annotations are less time consuming to generate than 3D annotations, as are sparse annotations compared to dense annotations. To build a successful 3D instance segmentation model for neuropil, dense ground-truth training data is typically recommended. We developed a lightweight method to generate dense 3D instance segmentations rapidly by using sparse 2D annotations for training. The generated segmentations can be used as pseudo ground-truth to bootstrap and iteratively refine a dedicated 3D model. Our method consists of two main steps: sparse to dense in 2D, followed by dense 2D to dense 3D.

### Sparse to Dense

During training, random fields of views from the annotated image(s) were chosen, augmentations were applied, and target predictions were computed from the sparse annotations. The areas lacking annotations were masked out during the computation of the loss between the model’s output prediction and the target prediction. This masking restricted the supervision during learning to only the labeled areas. Thus, the model could infer predictions in the unlabeled areas using what it learned from the labeled areas (Fig. 1a).

### 2D to 3D

We used a 2D network to generate dense 2D predictions for every section of a volume. These predictions were then stacked and used as input to a lightweight 3D network, pre-trained on synthetic 3D data, to output 3D boundaries (Fig. 1b). The synthetic 3D data is generated on-the-fly during training using image-processing and morphological operations, from which the simulated stacked 2D input and target 3D output are created (Supp. Fig 3, Supp Note E). In practice, images from different modalities could adapt the same model since it only uses stacked 2D predictions as input and not the stacked raw images.

### Networks

All network architectures, training pipelines, and parameter values explored in the post-processing grid-searches are described in detail in the Supplementary Notes D, E, and Supplementary Tables 3–8. GPU nodes with NVIDIA A100s were used for training and inference. Texas Advanced Computing Center (TACC)’s Launcher software was used to run the post-processing and evaluation jobs on TACC’s Lonestar6^38^. Hyperparameter optimization was done through a grid search as described in Supplementary Note B. Importantly, we note a computing cluster is not needed to generate new 3D segmentations with our lightweight algorithms. We report the parameter counts and vRAM used by the different networks in Supplementary Table 2.

We chose 2D and 3D U-NETs to learn dense cell boundaries, embeddings like LSDs, or both^13,39^. We used direct-neighbor affinity graphs (affinities) to represent the plasma membrane boundaries by capturing the connectivity (or affinity value) of every pixel to its immediate neighbors^3^. This representation, compared to binary boundary representations, resolves ambiguities when partitioning voxels into different instances, especially when the instances share boundaries.

For bootstrapping a neuropil segmentation model from pseudo ground-truth data, we chose the 3D Multi-task LSD (3D MTLSD) U-NET. For small volumes, the addition of LSDs as an auxiliary learning task for affinities achieves comparable accuracy to the current state of the art, while being 100x more computationally efficient^7^. The higher speed of these MTLSD networks is invaluable in this context given the potential need for repeated iterations of generating and proofreading segmentations.

### Post-processing

We used our published methods^5,7,8^ to generate dense 3D instance segmentations from affinities. First, we thresholded predicted affinities to generate a binary mask, from which we computed a distance transform and identified local maxima. We used the maxima as seeds for a watershed algorithm to generate an over-segmentation (resulting in supervoxels). Each supervoxel center of mass was stored as a node with coordinates in a region adjacency graph. All nodes of touching supervoxels were connected by edges and added to this graph. In a subsequent agglomeration step, edges were hierarchically merged using the underlying predicted affinities as weights, in order of decreasing affinity.

## Supplementary Material

Supplement 1
