# Supplementary material for: Sparse Annotation is Sufficient for Bootstrapping Dense Segmentation
Source: Res Sq. 2024 Nov 14:rs.3.rs-5339143. Preprint. [Version 1] doi: 10.21203/rs.3.rs-5339143/v1 (PMC11601847; doi:10.21203/rs.3.rs-5339143/v1)
Supplement: Supplement 1 [file NIHPPRS5339143V1-supplement-1.pdf]

Supplementary Material

A. Sparsities

We define a *sparsity* as an amount of manual annotations. This sparse amount of manual annotations is used to train networks with a weighted loss function to infer dense predictions.

We sought to explore how sparse manual annotations can be in this context of neuron segmentation in electron microscopy (EM). Our objective was to identify efficient strategies for generating ground truth labels that provide sufficient information for the network's learning, while minimizing the associated human annotation costs. We explored a total of 8 sparsities at the instance or object-level:

- 1 object
- 2 objects
- 2 adjacent objects
- 5 objects
- 10 objects
- 50 objects
- 100 objects
- All objects (dense)

For each of the above object-level sparsities, we included three additional disk sparsities, resulting in a total of 32 sparsities (Supp. Fig. 1). We included disk sparsities based on considerations from the local shape descriptors (LSDs); the regions thought to be most crucial for learning LSDs are concentrated around object boundaries and entirely within objects[1]. Under this assumption, sparsity could theoretically be represented by deliberately positioned incomplete objects (such as paint strokes). For example, placing a stroke on one side of an object, another stroke on the opposite side, and a stroke in the center could contain much of the necessary information to instruct a network about the presence of a smooth gradient inside an object and sharp transitions across boundaries. Unlabeled areas could then be considered unknown using a weighted loss function. Hence, for a disk sparsity of N disks, we selected N points within the field of view (FOV) and drew circles with a sufficient radius intersecting the labels.

The intersecting circle cuts off the labels in disk sparsities, leading to incorrect ground truth LSDs towards the center of objects where there should be a sharp gradient instead of a smooth or small gradient. We expected this discrepancy to adversely impact predictions and result in circular artifacts or false boundaries. However, we were surprised to observe that the network still predicted these regions correctly. This could be attributed to several factors: Firstly, we randomly sampled enough locations in batches that were deemed correct. An ablation study could involve limiting these

circles to always include incorrect boundaries. Secondly, the network might become confused in these regions due to conflicting signals from the "correct" regions. Consequently, the network regresses to predict approximately 0.5 in these areas, which happens to map to gray in RGB space, akin to the intentional design of the original LSDs.

B. Experiment and grid search

The experimental procedure in Fig. 2 is described here in more detail. For every dataset, sparsity, and repetition:

1. The 2D U-Net of the 2D→3D method was trained on the sparse 2D annotations of Volume 1. The three variations of the 2D U-Nets explored differ by output: 2D affinities, 2D LSDs, or both (2D MTLSD).
2. Inference was done with different iterations of the trained 2D→3D models to generate predictions of 3D affinities of Volume 2.
    a. Stacked 2D predictions are made with an iteration of the trained 2D U-Net. The predictions tested were: 2D affinities, 2D LSDs, 2D affinities (from MTLSD), 2D LSDs (from MTLSD). (4 variations)
    b. 3D affinities of Volume 2 are made with an iteration of the trained 3D U-Net of the 2D→3D method. All four strategies to generate synthetic 3D labels during training were explored. (4 variations)
3. Post-processing was done in a grid-fashion to generate 3D segmentations of Volume 2 from all different combinations of model iterations, network variations, and post-processing parameters. (Supp. Table 5)
4. All the generated segmentations of Volume 2 were evaluated for accuracy (Supp. Figs. 4-7.). The best segmentation was designated as the pseudo ground-truth training data for the untrained 3D MTLSD model without any proofreading.
5. The 3D MTLSD model was trained on the pseudo ground-truth training data of Volume 2.
6. Inference was done with different iterations of the trained 3D MTLSD model to generate predictions of 3D affinities of Volume 1.
7. Post-processing was done in a grid-fashion to generate 3D segmentations of Volume 1 from the different predictions. (Supp. Table 7,8)
8. All the generated segmentations of Volume 1 were evaluated for accuracy. (Fig. 3, Supp. Fig. 9 -13). Total time to segmentation starting from sparse annotation was estimated. The best segmentation in terms of accuracy was designated as the representative bootstrapped segmentation for the dataset, sparsity, and rep in question.

C. Evaluation metrics

We automatically generated skeletons for evaluation from ground-truth labels with the following steps. We used a watershed algorithm on computed ground-truth affinities to

generate an over-segmentation (resulting in supervoxels). Each supervoxel center of mass was stored as a node with position coordinates in a region adjacency graph. For each ground-truth mask, we computed the minimum spanning-tree of the nodes using the physical distance between nodes as the weight. We note that the skeletons are not true ground-truth as there may be edge cases with this method which could affect the accuracy.

We compare the accuracy of bootstrapped segmentations using the normalized, variation of information (VOI) metric and min-cut metric (MCM)[1–3]. VOI is the voxel-based measure of similarity between a segmentation and ground-truth labels. VOI reports the amount of split and merge errors. We note that VOI can be sensitive to slight differences in boundaries. At the same time, small topological changes might go unnoticed, which is especially problematic in fine neuropil. Nevertheless, VOI can be a good proxy for segmentation quality[1].

Quantifying segmentation accuracy in terms of proofreading effort required to correct it is more interpretable and relevant to optimize. False splits require only one interaction to merge. False merges can also be fixed with a few interactions[1,4,5]. The MCM measures the total number of split and merge edit operations needed to make a segmentation agree with ground-truth skeletons. We report the total edits per object, total edits per path length, total splits and total merges per object.

D. Network architectures

All networks were trained using gunpowder (https://funkelab.github.io/gunpowder/) and PyTorch, using the U-Net architecture implemented in (github.com/funkelab/funlib.learn.torch). Code for LSDs is available at (github.com/funkelab/lsd) and code for example 2D→3D networks and 3D MTLSD networks are available at (github.com/ucsdmanorlab/bootstrapper) and (github.com/ucsdmanorlab/sparsity_experiments)

The 3D MTLSD network used for bootstrapping was a 3D U-Net with two separate decoder heads for the two learning tasks: affinities and LSDs. The MTLSD U-Net consisted of three layers with downsampling factors [1,2,2]. The bottleneck and the adjacent layers have 2D convolution kernels. Thirteen initial feature maps were used with a multiplication factor of 6 between layers. The resulting data was further convolved and passed through a sigmoid activation to get from 13 output feature maps from each decoder head to 3 (3D affinities) and 10 (3D LSDs).

The 2D U-Nets used in the 2D→3D method consisted of three layers and were downsampled by a factor of [2,2] in all layers. Twelve initial feature maps were used, and features were multiplied by a factor of 6 between layers. The resulting data was further convolved and passed through a sigmoid activation to get from 12 output feature maps to either 2 (2D affinities), 6 (2D LSDs) or 8 feature maps (2D MTLSD).

The lightweight 3D U-Nets in the 2D→3D method consisted of two layers and were downsampled by a factor of [1,2,2]. The convolution kernel sizes for the first downsampling and last upsampling layers are [2,3,3] and [1,3,3] for the other layers. 5 initial feature maps were used, and features were multiplied by a factor of 5 between layers. The resulting data was further convolved and passed through a sigmoid activation to get from 5 output feature maps to 3 (3D affinities). All networks used a mean-squared error loss, minimized with an Adam optimizer.

E. Training pipelines

For all sparse 2D and 3D networks and the 3D MTLSD network used for bootstrapping, each training batch was randomly picked from the available sections or volume. For each batch, the raw data was first normalized and padded with zeros. Labels were padded with the maximum padding required to contain at least 0.01% (10% for 3D MTLSD, since there are more pseudo-GT labels available) of labeled ground-truth data within the image assuming a worst case rotation of 45 degrees. Data was randomly sampled and augmented with elastic transformations, random mirrors and transposes, gaussian blur, and intensities (see Supplementary Tables 3 and 6 for augmentation hyper-parameters used for the networks). For the networks with affinities as an output, a scale array was created to balance loss between target affinity labels.

For the lightweight 3D networks in the 2D→3D method, each training batch begins as a 3D array of zeros. Synthetic 3D labels are randomly grown using the strategies listed below and illustrated in Supp. Fig 3. Labels were then augmented with elastic transformations and random mirrors and transposes, after which they were used to simulate stacked 2D affinities or LSDs. The stacked 2D affinities or LSDs were then augmented with random noise, intensities and gaussian blur to simulate realistic stacked 2D predictions. Finally, target 3D affinities were computed, and a scale array was created to balance loss between class labels.

Synthetic 3D Labels Generation

A. N many randomly chosen voxels in the array of zeros get set to 1, where N is a random integer between 25 and 50. The speckled array is relabeled such that each labeled voxel has a unique integer ID. The labels are grown outward by D pixels without overlapping other labels, where D is another random integer between 25 and 40. The ID of the background label is bumped to a non-zero and unique integer ID.
B. Like A, but the speckled binary array is dilated section-wise with the binary structure of a 2x2 square or a disk binary structure with a random radius between 1 and 5. The binary array is then labeled such that each foreground instance has a unique integer ID. The uniquely labeled objects are then expanded into unoccupied spaces using a distance transform of the background.

C. A gaussian filter with sigma=5 is applied to an array of random float values to obtain peaks. The peaks are accentuated with a maximum filter with a size of 10 pixels and maxima are identified as seeds. Watershed is then applied to the peaks to grow labels from the seeds.
D. An equal mix of the above three strategies.

Dataset Name	Imaging modality	Content	Resolution (nm/px, ZYX)	Size (pixels, ZYX)	Number of objects
HARRIS-15[6]	TEM	*Rattus* hippocampal neuropil	(50, 2, 2), downscaled to (50, 8, 8)	(101,900,927)	444
				(70,674,504)	181
FIB-25[7]	FIBSEM	*Drosophila* optic medulla neuropil	(8, 8, 8)	(520,520,520)	1690
				(520,520,520)	2031
CREMI-C[8]	SSTEM	*Drosophila* calyx neuropil	(40, 4, 4) Downscaled to (40, 8, 8)	(62,625,625)	546
				(63,625,625)	647
CREMI-B[8]		*Drosophila* axon tract		(62,625,625)	450
				(63,625,625)	581
CREMI-A[8]		*Drosophila* axon tract		(63,625,625)	333
				(62,625,625)	312
EPI[9]	LM	*Arabidopsis* epithelial cells	(235, 75, 75)	(318,960,953)	3333
				(248,791,667)	1053

Supp. Table 1 | Overview of datasets. Dataset rows show Volume 1 above Volume 2. A size filter of 500 pixels was applied to the labels before counting objects.

Network	Number of trainable parameters	vRAM usage
2D U-Net (affs)	88,886,116	2862MiB
2D U-Net (lsds)	88,886,220	2586MiB
2D→3D U-Net	264,206	634MiB
3D U-Net (MTLSD)	187,077,748	6186MiB

Supp. Table 2 | Number of trainable parameters and vRAM usage for the tested models.

Parameter	Value
Input feature maps	12
Layer feature map scale	6
Downsampling factors	[[2, 2], [2, 2], [2, 2]]
Input shape	[196, 196]
Output shape	[104, 104]
Loss	Weighted MSE
Optimizer	Adam
Learning rate	0.5 × 10^-4
β1	0.9
β2	0.999
ε	1 × 10^-8
Iterations	20,000

Augmentation	Parameter	Value
Elastic	Control point spacing	(8, 8)
	Jitter Sigma	(2, 2)
	Subsample	4
Rotation	Axis	x, y
	Angle	in [0, 2π]
Mirror	Axes	x, y
Transpose	Axes	x, y
Noise	Mode	Gaussian
Intensity	Scale	in [0.9, 1.1]
	Shift	in [−0.1, 0.1]
Blur	Sigma	In [0.0, 1.5]

Supp. Table 3 | Training parameters and augmentations of 2D networks in the 2D→3D method on HARRIS-15.

Parameter	Value
Input feature maps	5
Layer feature map scale	5
Downsampling factors	[[1, 2, 2], [1, 2, 2]]
Kernel sizes down	[[2, 3, 3], [2, 3, 3]],<br>[[1, 3, 3], [1, 3, 3]],<br>[[1, 3, 3], [1, 3, 3]]
Kernel sizes up	[[1, 3, 3], [1, 3, 3]],<br>[[2, 3, 3], [2, 3, 3]],
Input shape	[10, 148, 148]
Output shape	[6, 108, 108]
Loss	Weighted MSE
Optimizer	Adam
Learning rate	0.5 × 10^-4
β1	0.9
β2	0.999
ε	1 × 10^-8
Iterations	20,000

Supp. Table 4 | Training parameters of the lightweight 3D networks in the 2D→3D method on HARRIS-15.

Parameter	Values	Number of variations
Synthetic 3D labels generation	A, B, C, D	4
3D affinities iteration	{5000, 10000, 15000, 20000} (2D U-Net)<br>x<br>{5000, 10000, 15000, 20000} {3D U-Net)	16
Rep	rep_1, rep_2, rep_3	3
Sparsity	10min_paint_{2d,3d}<br>+<br>paint_{2d, 3d}<br>+<br>{disk_{0,1,2,3}} x<br>obj_{001,002,002a,005,010,100,dense}}<br>+<br>3D_dense	33
Watershed minimum seed distance	10	1
Hierarchical merge function	“mean”, “hist_quant_50”, “hist_quant_75”	3
Total parameter grid size		19008

Supp. Table 5: Post-processing parameter grid explored for generating 2D→3D segmentations on HARRIS-15.

Parameter	Value
Input feature maps	13
Layer feature map scale	6
Downsampling factors	[[1, 2, 2], [1, 2, 2], [1, 2, 2]]
Kernel sizes down	[[3, 3, 3], [3, 3, 3]],<br>[[3, 3, 3], [3, 3, 3]],<br>[[1, 3, 3], [1, 3, 3]],<br>[[1, 3, 3], [1, 3, 3]]
Kernel sizes up	[[1, 3, 3], [1, 3, 3]],<br>[[3, 3, 3], [3, 3, 3]],<br>[[3, 3, 3], [3, 3, 3]]
Input shape	[20, 196, 196]
Output shape	[4, 104, 104]
Loss	Weighted MSE
Optimizer	Adam
Learning rate	0.5 × 10^-4
β1	0.9
β2	0.999
ε	1 × 10^-8
Iterations	50,000

Augmentation	Parameter	Value
Elastic	Control point spacing	(8, 50, 50)
	Jitter Sigma	(0, 2, 2)
	Subsample	4
	Scale interval	(0.75, 1.25)
Rotation	Axis	x, y
	Angle	in [0, 2π]
Mirror	Axes	x, y, z
Transpose	Axes	x, y
Noise	Mode	Gaussian
Intensity	Scale	in [0.9, 1.1]
	Shift	in [−0.1, 0.1]
Blur	Sigma	In [0.0, 1.5]

Supp. Table 6: Training parameters and augmentations of 3D networks on HARRIS-15.

Parameter	Values	Number of variations
Predicted affinities iteration	20000, 35000, 50000	3
Rep	rep_1, rep_2, rep_3	3
Pseudo GT network	Affinities,<br>LSDs,<br>Affinities from MTLSD,<br>LSDs from MTLSD	4
Pseudo GT Sparsities	10min_paint_{2d,3d}<br>+<br>paint_{2d, 3d}<br>+<br>obj_{001,002,002a,005,010,100,2D_dense, 3D_dense}	13
Normalize affinities	False	1
Watershed minimum seed distance	10	1
Watershed boundary mask	True	1
Hierarchical merge function	“mean”	1
Total parameter grid size		468

Supp. Table 7 | Post-processing parameter grid with VOI and MCM explored for generating bootstrapped segmentations on HARRIS-15.

**Parameter**	**Values**	**Number of variations**
Predicted affinities iteration	20000, 35000, 50000	3
Rep	rep_1, rep_2, rep_3	3
Pseudo GT network	Affinities,<br>LSDs,<br>Affinities from MTLSD,<br>LSDs from MTLSD	4
Pseudo GT Sparsities	10min_paint_{2d,3d}<br>+<br>paint_{2d, 3d}<br>+<br>obj_{001,002,002a,005,010,100,2D_dense, 3D_dense, GT_dense}	13
Normalize affinities	False, True	2
Watershed minimum seed distance	5, 10, 15, 20	4
Watershed boundary mask	True, False	2
Hierarchical merge function	'hist_quant_10', 'hist_quant_25', 'hist_quant_50', 'hist_quant_75', 'hist_quant_90', 'mean'	6
Total parameter grid size		44928

Supp. Table 8 | Post-processing parameter grid with VOI explored for generating bootstrapped segmentations on HARRIS-15.

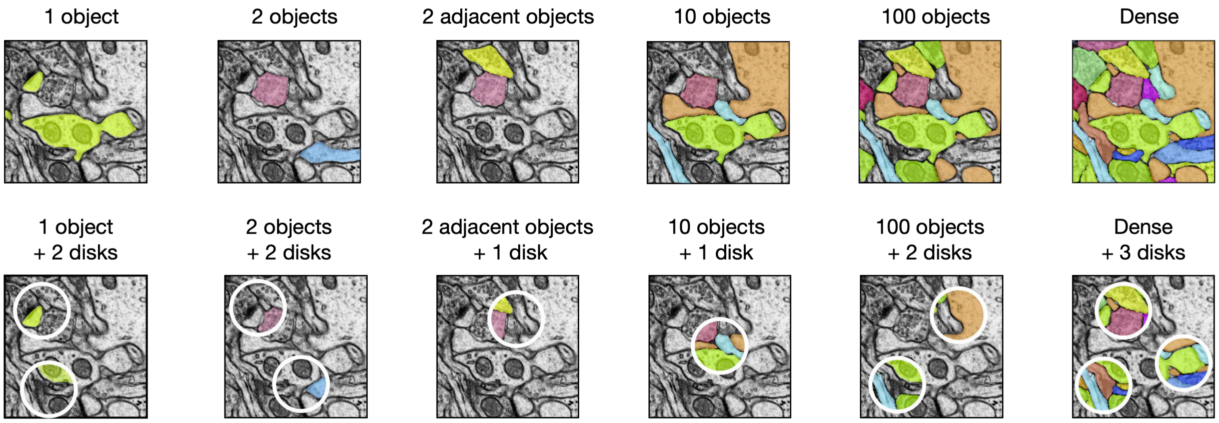

Supp. Fig. 1 | Example simulated sparsity regimes. Example HARRIS 15 image and ground-truth labels in training batches for different simulated sparsity levels which involve object-level ablations and disk-selections (white circles).

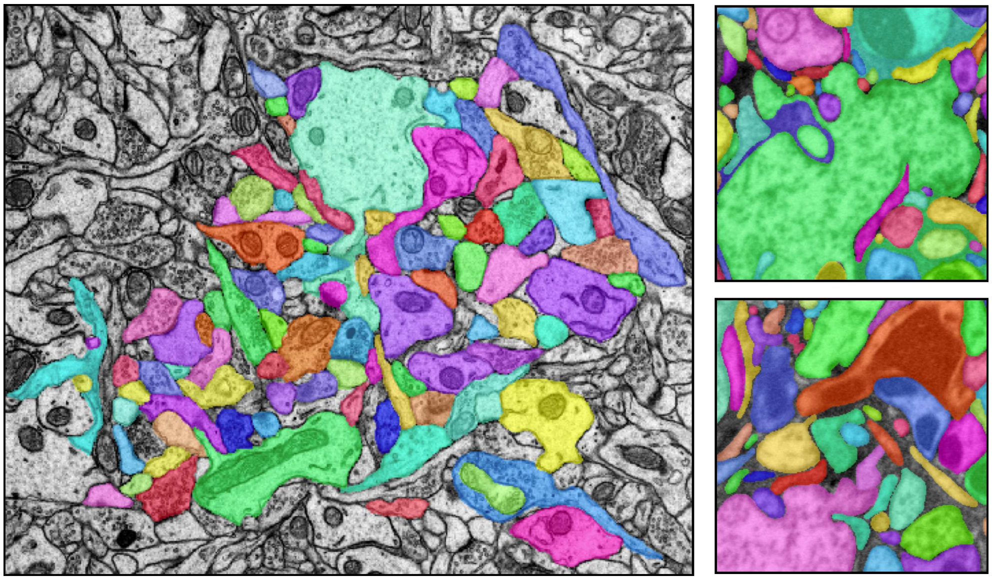

Supp. Fig. 2 | Example non-expert 2D annotations. Example image and overlaid non-expert annotations for HARRIS-15 (left) and FIB-25 (right) made in 30 minutes.

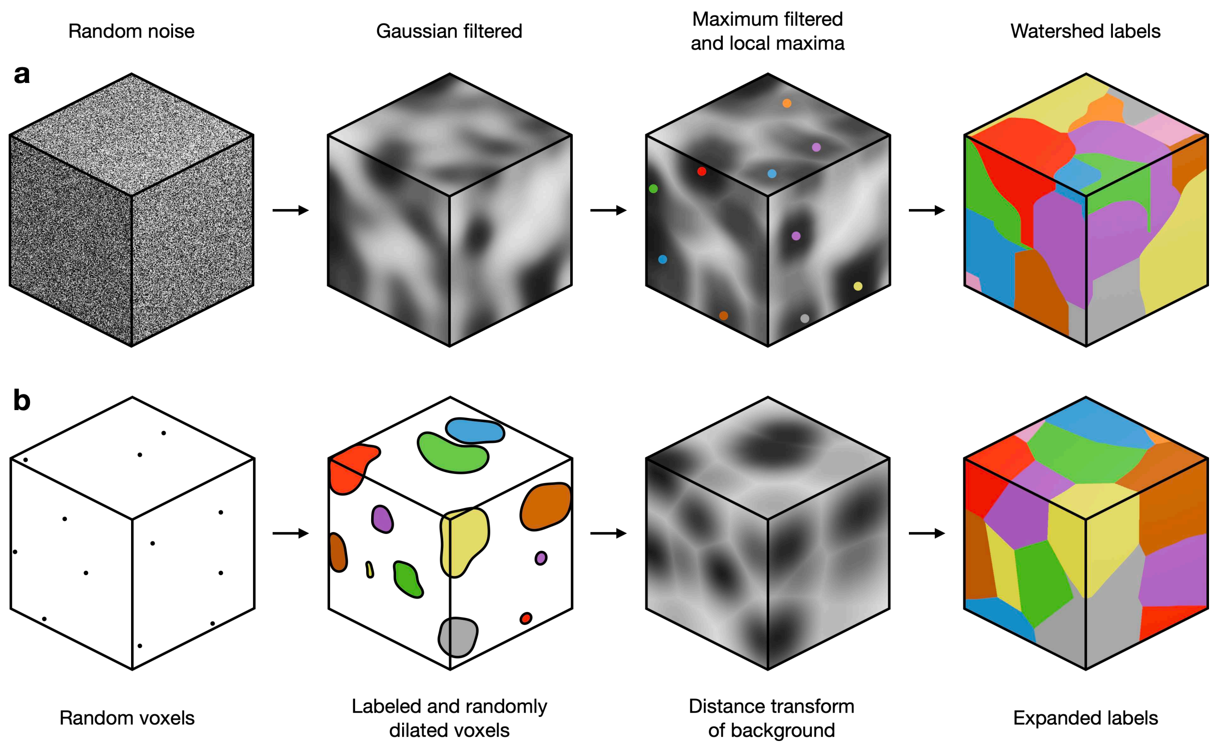

Supp. Fig. 3 | Synthetic 3D labels generation. Two different methods of generating a synthetic 3D labels array. **a,** A gaussian filter is applied on a 3D array of random values to obtain peaks, from which local maxima are obtained using a maximum filter. The maxima are labeled and used as seeds for watershed on the inverted peaks. **b,** A random sample of points in an empty 3D array are randomly dilated and labeled. The Euclidean distance transform of the background is used to expand the labels into unoccupied regions.

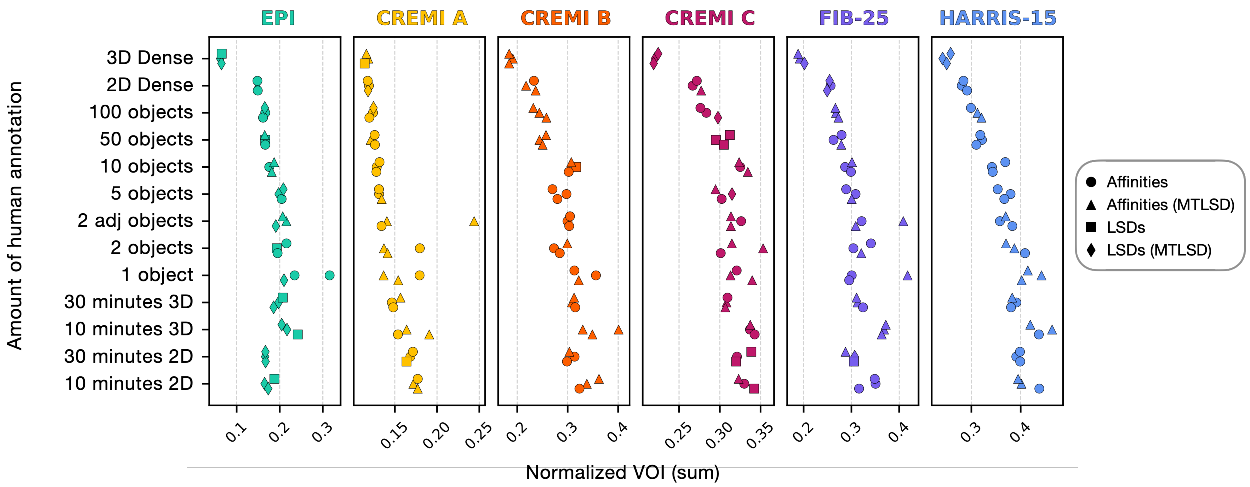

Supp. Fig. 4 | Quantitative results of 2D→3D models. Normalized Variation of Information (VOI) sum scores, lower scores are better. Scores were computed by comparing ground-truth labels to segmentations produced by the 2D→3D method. Plot includes best scores from parameter grid-searches, for each dataset and repetition versus the sparse paintings and object-level sparsities and one (1) intersecting disk. Dense refers to un-ablated labels. 2D (or 3D) Dense refers to the segmentation generated by the 2D→3D method (or a 3D model) after training on un-ablated 2D (or 3D) labels. Marker shapes indicate the best performing 2D→3D strategy.

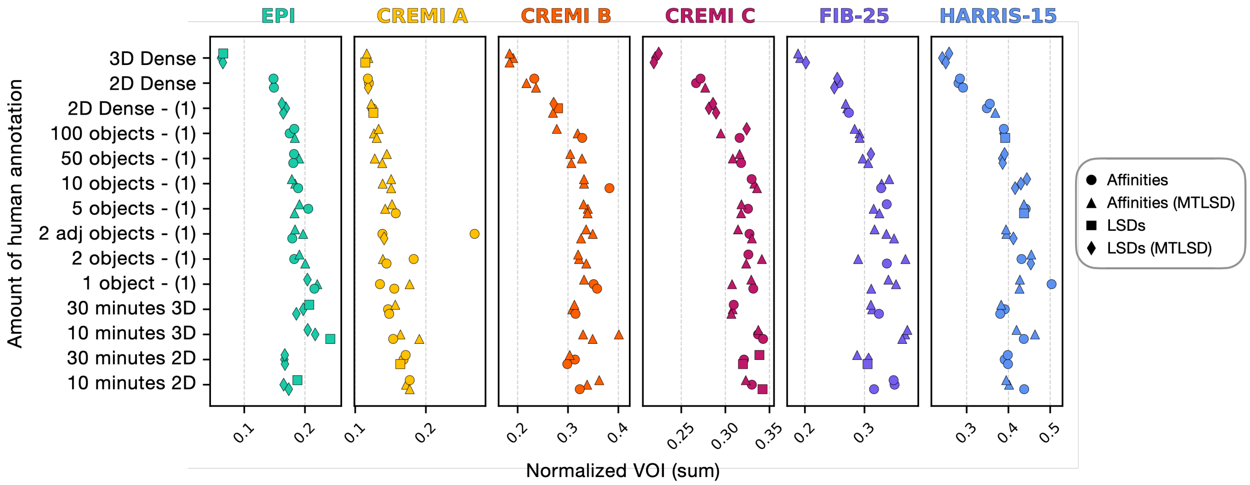

Supp. Fig. 5 | Quantitative results of 2D→3D models with one intersecting disk. Normalized Variation of Information (VOI) sum scores, lower scores are better. Scores were computed by comparing ground-truth labels to segmentations produced by the 2D→3D method. Plot includes best scores from parameter grid-searches, for each dataset and repetition versus the sparse paintings and object-level sparsities and one (1) intersecting disk. Dense refers to un-ablated labels. 2D (or 3D) Dense refers to the segmentation generated by the 2D→3D method (or a 3D model) after training on un-ablated 2D (or 3D) labels. Marker shapes indicate the best performing 2D→3D strategy.

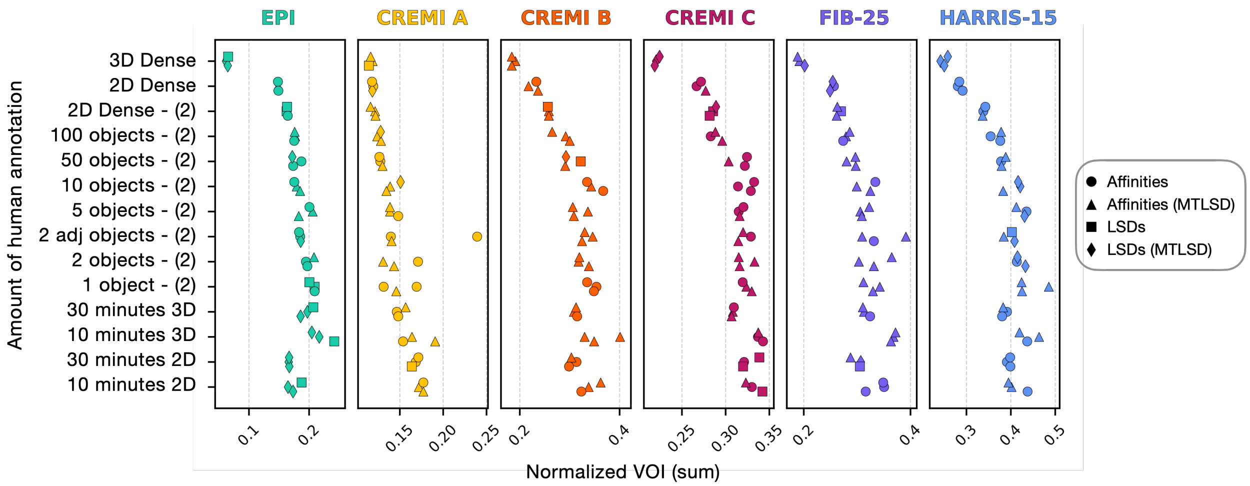

Supp. Fig. 6 | Quantitative results of 2D→3D models with two intersecting disks. Normalized Variation of Information (VOI) sum scores, lower scores are better. Scores were computed by comparing ground-truth labels to segmentations produced by the 2D→3D method. Plot includes best scores from parameter grid-searches, for each dataset and repetition versus the sparse paintings and object-level sparsities and two (2) intersecting disk. Dense refers to un-ablated labels. 2D (or 3D) Dense refers to the segmentation generated by the 2D→3D method (or a 3D model) after training on un-ablated 2D (or 3D) labels. Marker shapes indicate the best performing 2D→3D strategy.

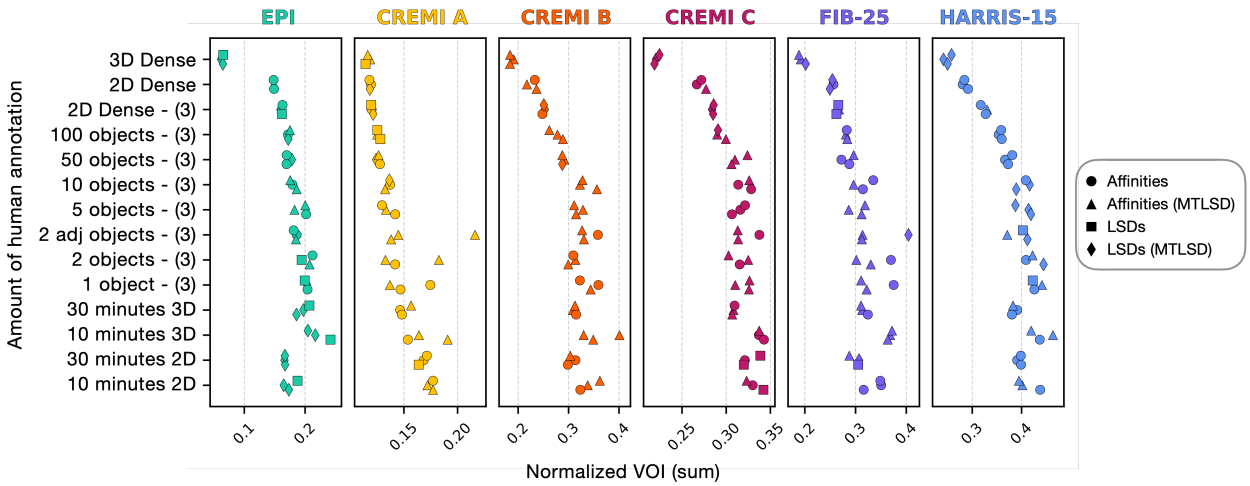

Supp. Fig. 7 | Quantitative results of 2D→3D models with three intersecting disks. Normalized Variation of Information (VOI) sum scores, lower scores are better. Scores were computed by comparing ground-truth labels to segmentations produced by the 2D→3D method. Plot includes best scores from parameter grid-searches, for each dataset and repetition versus the sparse paintings and object-level sparsities and three (3) intersecting disk. Dense refers to un-ablated labels. 2D (or 3D) Dense refers to the segmentation generated by the 2D→3D method (or a 3D model) after training on un-ablated 2D (or 3D) labels. Marker shapes indicate the best performing 2D→3D strategy.

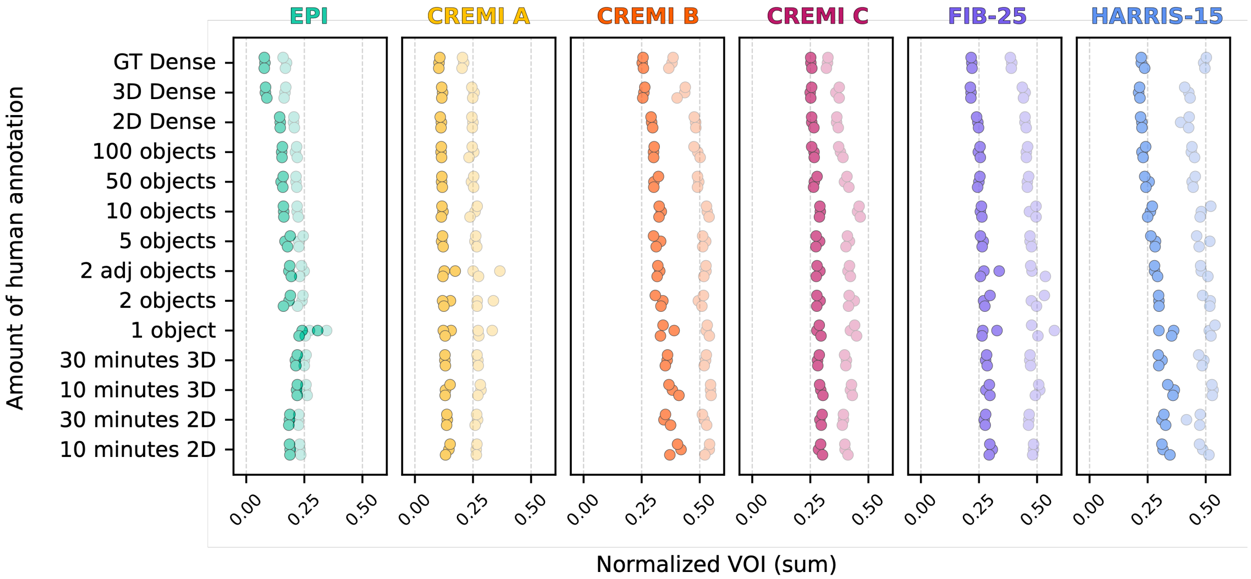

Supp. Fig. 8 | Effect of total parameter grid size on quantitative results. Lower scores are better. Normalized Variation of Information (VOI) sum scores of bootstrapped 3D models with parameter grid size per dataset of 44928 runs (opaque points) 468 runs (transparent points). See Supp. Table 6 for parameters used.

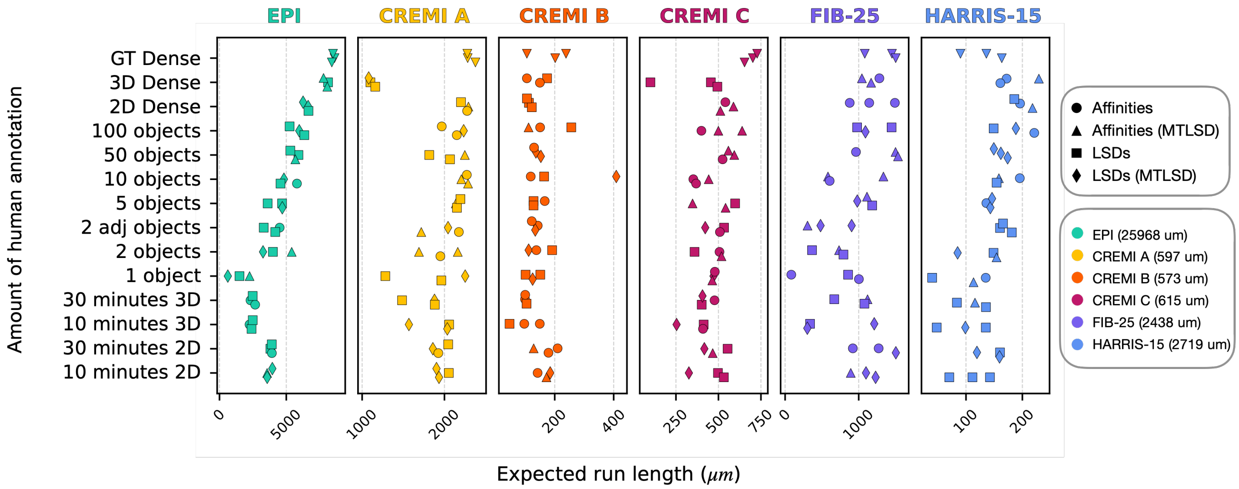

Supp. Fig. 9 | Expected run length of bootstrapped 3D segmentations. Higher scores are better. Marker shapes show best 2D→3D strategy, with three separate tests per amount of human annotation (three dots of same color per row). The total path length in microns of the dense ground-truth skeletons for each training volume is indicated in parenthesis in the legend. A 2D→3D model, trained on different amounts of initial ground truth (sorted from bottom to top of the y-axis in order of increasing human annotation effort), created dense segmentations of a test volume (pseudo ground-truth). Scores compare real ground-truth to outputs from a 3D model trained on the pseudo ground-truth. 2D (or 3D) Dense refers to training the initial 2D→3D (or 3D) model on all available 2D (or 3D) annotations to generate pseudo ground-truth. GT Dense refers to directly training a 3D model on ground-truth annotations of the test volume to generate the segmentation of the initial training volume.

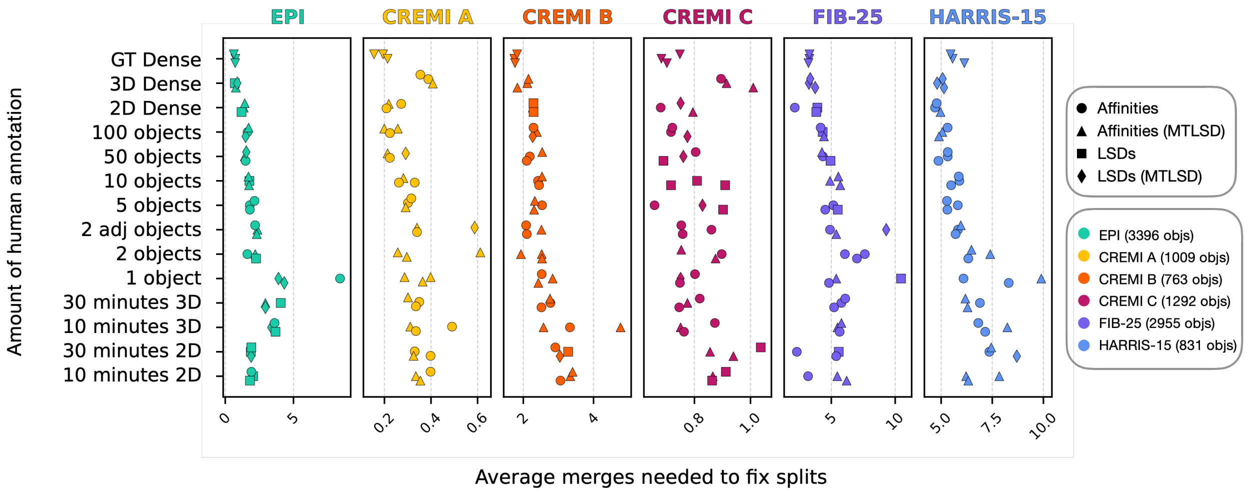

Supp. Fig. 10 | Average number of merges needed to fix splits for bootstrapped 3D segmentations. Lower scores are better. Marker shapes show best 2D→3D strategy, with three separate tests per amount of human annotation (three dots of same color per row). The total number of evaluation ground-truth objects for each training volume is indicated in parenthesis in the legend. A 2D→3D model, trained on different amounts of initial ground truth (sorted from bottom to top of the y-axis in order of increasing human annotation effort), created dense segmentations of a test volume (pseudo ground-truth). Scores compare real ground-truth to outputs from a 3D model trained on the pseudo ground-truth. 2D (or 3D) Dense refers to training the initial 2D→3D (or 3D) model on all available 2D (or 3D) annotations to generate pseudo ground-truth. GT Dense refers to directly training a 3D model on ground-truth annotations of the test volume to generate the segmentation of the initial training volume.

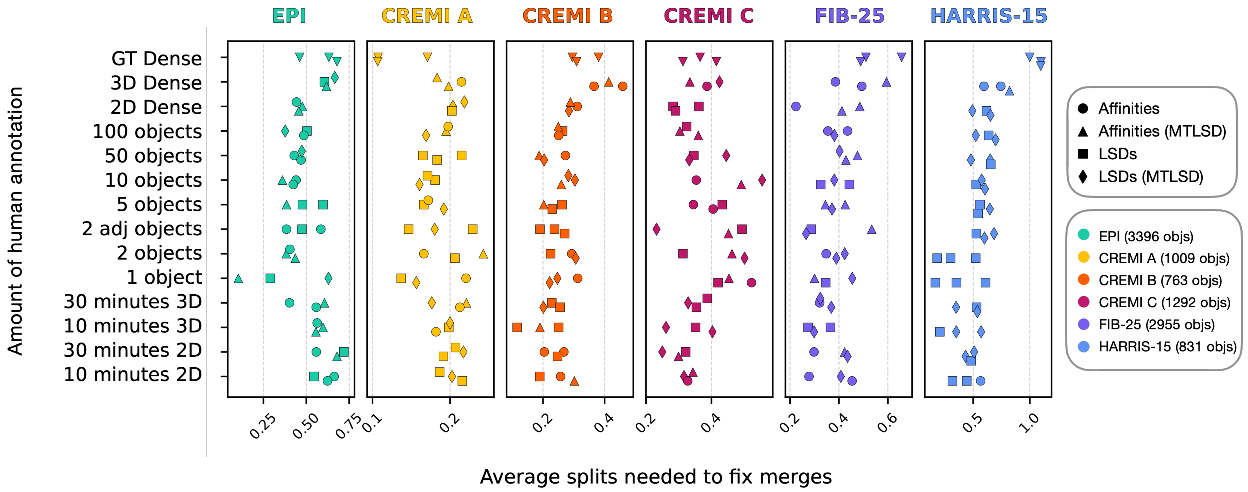

Supp. Fig. 11 | Average number of splits needed to fix merges for bootstrapped 3D segmentations. Lower scores are better. Marker shapes show best 2D→3D strategy, with three separate tests per amount of human annotation (three dots of same color per row). The total number of evaluation ground-truth objects for each training volume is indicated in parenthesis in the legend. A 2D→3D model, trained on different amounts of initial ground truth (sorted from bottom to top of the y-axis in order of increasing human annotation effort), created dense segmentations of a test volume (pseudo ground-truth). Scores compare real ground-truth to outputs from a 3D model trained on the pseudo ground-truth. 2D (or 3D) Dense refers to training the initial 2D→3D (or 3D) model on all available 2D (or 3D) annotations to generate pseudo ground-truth. GT Dense refers to directly training a 3D model on ground-truth annotations of the test volume to generate the segmentation of the initial training volume.

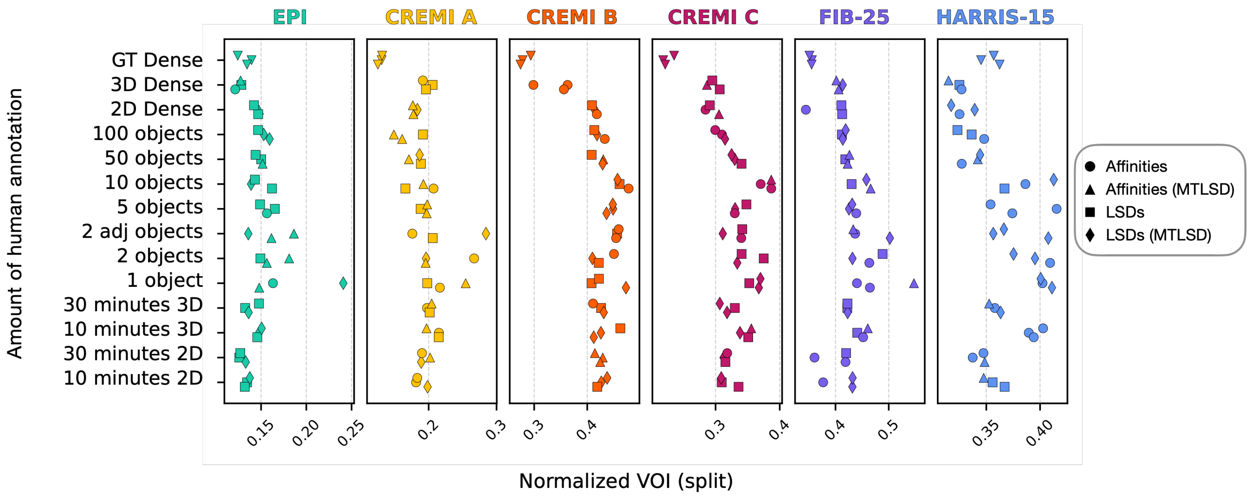

Supp. Fig. 12 | Normalized VOI split for bootstrapped 3D segmentations. Lower scores are better. Marker shapes show best 2D→3D strategy, with three separate tests per amount of human annotation (three dots of same color per row). A 2D→3D model, trained on different amounts of initial ground truth (sorted from bottom to top of the y-axis in order of increasing human annotation effort), created dense segmentations of a test volume (pseudo ground-truth). Scores compare real ground-truth to outputs from a 3D model trained on the pseudo ground-truth. 2D (or 3D) Dense refers to training the initial 2D→3D (or 3D) model on all available 2D (or 3D) annotations to generate pseudo ground-truth. GT Dense refers to directly training a 3D model on ground-truth annotations of the test volume to generate the segmentation of the initial training volume.

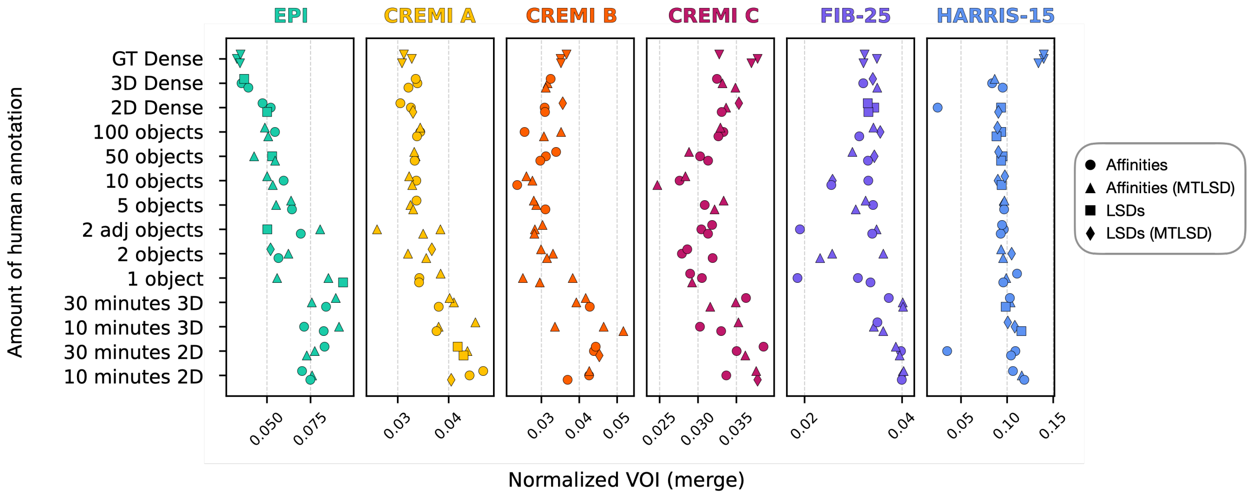

Supp. Fig. 13 | Normalized VOI merge for bootstrapped 3D segmentations. Lower scores are better. Marker shapes show best 2D→3D strategy, with three separate tests per amount of human annotation (three dots of same color per row). A 2D→3D model, trained on different amounts of initial ground truth (sorted from bottom to top of the y-axis in order of increasing human annotation effort), created dense segmentations of a test volume (pseudo ground-truth). Scores compare real ground-truth to outputs from a 3D model trained on the pseudo ground-truth. 2D (or 3D) Dense refers to training the initial 2D→3D (or 3D) model on all available 2D (or 3D) annotations to generate pseudo ground-truth. GT Dense refers to directly training a 3D model on ground-truth annotations of the test volume to generate the segmentation of the initial training volume.

References

1. Sheridan, A. *et al.* Local shape descriptors for neuron segmentation. *Nat. Methods* **20**, 295–303 (2023).
2. Meilă, M. Comparing clusterings—an information based distance. *J. Multivar. Anal.* **98**, 873–895 (2007).
3. Vinh, N. X., Epps, J. & Bailey, J. Information Theoretic Measures for Clusterings Comparison: Variants, Properties, Normalization and Correction for Chance. *J. Mach. Learn. Res.* **11**, 2837–2854 (2010).
4. Zhao, T., Olbris, D. J., Yu, Y. & Plaza, S. M. NeuTu: Software for Collaborative, Large-Scale, Segmentation-Based Connectome Reconstruction. *Front. Neural Circuits* **12**, 101 (2018).
5. Dorkenwald, S. *et al.* FlyWire: online community for whole-brain connectomics. *Nat. Methods* **19**, 119–128 (2022).
6. Harris, K. M. *et al.* A resource from 3D electron microscopy of hippocampal neuropil for user training and tool development. *Sci. Data* **2**, 150046 (2015).
7. Takemura, S. *et al.* Synaptic circuits and their variations within different columns in the visual system of Drosophila. *Proc. Natl. Acad. Sci.* **112**, 13711–13716 (2015).
8. CREMI. https://cremi.org/.
9. Wolny, A. *et al.* Accurate and versatile 3D segmentation of plant tissues at cellular resolution. *eLife* **9**, e57613 (2020).
